# A Novel *Trichinella spiralis* Galectin Strengthens the Macrophage ADCC Killing of Larvae via Driving M1 Polarization

**DOI:** 10.3390/ijms252010920

**Published:** 2024-10-10

**Authors:** Minmin Weng, Ru Zhang, Zhaoyu Zhang, Jinyi Wu, Wenwen Zheng, Qiqi Lu, Shaorong Long, Ruodan Liu, Zhongquan Wang, Jing Cui

**Affiliations:** Department of Parasitology, School of Basic Medical Sciences, Zhengzhou University, Zhengzhou 450001, China; wengminmin2022@163.com (M.W.); zhangru2020@126.com (R.Z.); zhangzhaoyu0000@126.com (Z.Z.); wujinyi202309@163.com (J.W.); zhengwenwen000@126.com (W.Z.); luqiqi_lqq@163.com (Q.L.); longshaorong@126.com (S.L.); liuruodan2006@126.com (R.L.)

**Keywords:** *Trichinella spiralis*, galectin, macrophage M1 polarization, ADCC, NF-κB

## Abstract

Galectin recognizes β-galactosides through its carbohydrate recognition domains (CRDs). This study aimed to determine the biological features of a novel *Trichinella spiralis* galectin (galactoside-binding lectin family protein, TsGLFP) and its role in driving macrophage M1 polarization and enhancing ADCC killing of larvae. TsGLFP belongs to the galectin family and has two CRDs. The complete TsGLFP cDNA sequence was cloned and then expressed in *Escherichia coli* BL21. The results of qPCR, Western blot, and indirect immunofluorescence tests (IIFTs) revealed that TsGLFP was expressed in various stages of *T. spiralis* worms and principally localized at the cuticle and around the female embryos of the nematode. rTsGLFP had the function of agglutinating mouse erythrocytes, and this agglutination activity could be inhibited by lactose. After the mouse macrophage RAW264.7 was incubated with rTsGLFP, the expression level of the M1 genes (iNOS, IL-6, and TNF-α) and NO production were obviously increased. After incubating macrophages with rTsGLFP, there was a noticeable rise in the expression levels of p-IκB-α and p-NF-κB p65. Additionally, rTsGLFP enhanced the macrophage’s ability to kill newborn larvae by ADCC cytotoxicity. When the macrophages were pretreated with the specific p-NF-κB p65 inhibitor PDTC, and then stimulated with rTsGLFP, the expression levels of iNOS, NO, and p-NF-κB p65 and the macrophages’ ADCC cytotoxicity were distinctly decreased. These findings indicated that rTsGLFP enhanced the macrophage ADCC killing of larvae by driving M1 polarization through activating the NF-κB pathway.

## 1. Introduction

*Trichinella* spp. is a kind of worldwide zoonotic parasitic nematode, and its natural hosts include mammals, rodents, amphibians, reptiles, and birds [1]. Human trichinellosis is caused by eating raw or poorly cooked meat of domestic or wild animals infected with *Trichinella* muscle larvae (MLs) [2,3,4]. In China, eight trichinellosis outbreaks consisting of 479 cases and 2 deaths were recorded between 2009 and 2020, and 7 of 8 outbreaks (87.50%) were caused by the ingestion of raw or undercooked swine pork [5]. Pork is still the primary source of *Trichinella* infection. Trichinellosis is an important foodborne zoonosis, which is not only a crucial public health problem but also a risk to meat safety [6]. However, it is hard to eradicate *Trichinella* spp. infection in animals for human consumption due to its broad spectrum of natural hosts and ineffective preventive vaccines [7,8]. Therefore, it is imperative to understand the mechanisms of survival and immune evasion of the nematode in the host to develop a new control strategy of *Trichinella* infection in animals [9,10,11].

*Trichinella spiralis* has a complicated lifecycle, which begins with the ingestion of encapsulated muscle larvae (MLs) in contaminated meat. The MLs are released from their capsule following gastric fluid digestion and then develop into intestinal infectious larvae (IILs) under the stimulation of bile and enteral contents. The IILs intrude into the gut epithelium where they undergo four molts to develop into adult worms (AWs) [12,13]. After copulation, pregnant females produce newborn larvae (NBLs). The NBLs enter the skeletal muscles through blood circulation and induce the infected muscle cells to become their own nutritional cells; then collagen capsules form around the MLs, thus finalizing the lifecycle [14].

*Trichinella spiralis* infection usually causes Th2-type immunity, as characterized by elevated levels of Th2 cytokines and IgE, and the infiltration of eosinophils, basophils, and mast cells [15]. During the intestinal stage of *T. spiralis* infection, the Th1 immune response is first dominant, followed by the IILs’ development in adults and the production of many NBLs. The Th2 immune response gradually strengthens and dominates, which plays a vital role in damaging and eliminating the parasite from the host [16,17,18]. Previous studies indicated that crude antigens from *T. spiralis* AWs prevented ovalbumin-induced airway inflammation by activating Treg cells, reducing eosinophil infiltration and specific IgE and IL-4 levels, raising the production of IL-10 and TGF-β [19]. According to recent research, *T. spiralis* antigens attenuate macrophage infiltration in the adipose tissues of obese mouse, reduce Th1 and Th17 cytokine expression levels, and boost Th2 cytokine expression, thereby improving the chronic inflammation of obese mice [20]. By eliciting a Th2-type response with M2 macrophages and raising IL-10 expression, the pretreatment of mice with a recombinant *T. spiralis*-derived serine protease inhibitor (rTsSPI) reduced colitis while reducing neutrophil recruitment and TNF-α expression [21]. However, the mechanism of immune response and escape evoked by infection with *T. spiralis* remains incompletely understood at this time.

Lectin is a kind of protein agglutinating erythrocytes and binding reversibly with carbohydrates present in the cells. Lectin has sugar-binding sites which are also called carbohydrate recognition domains (CRDs), and the binding could be suppressed by one or more carbohydrates [22,23,24]. Based on the molecular structure, animal lectin is classified into C (containing selectin), S (galectin), P, and I types. Galectin, a family of the S type, is characterized by carrying a CRD and having a specific affinity to β-galactoside. Galectin has a significant function in inflammation reactions, immune responses, and signaling pathways. Extracellular galectin regulates the activity of interacting cells by binding to glycosylation receptors on the cells, while intracellular galectin interacts with others [25,26]. Galectin also plays a crucial role in the intrusion and pathogenesis of some parasite infections [27]. The galectin-1 expressed on *Angiostrongylus cantonensis* L5 larval epidermis promoted nematode parasitism [28], and *Brugia malayi*-secreted galectin-2 modulated immune responses and induced the Th1 cell apoptosis of the host [29]. The galectin from *Wuchereria bancrofti* mainly induces immune responses dominated by IgG and IgM responses [30]. A recombinant *Haemonchus contortus* galectin domain containing protein enhanced the expression of IL-4 and IL-9 in goat peripheral blood mononuclear cells (PBMCs) and induced Th2 and Th9 cell differentiation in a dose-dependent manner [31]. However, the literature contains only a few reports on the characteristics and role of *T. spiralis*-derived galectin [32].

Our previous laboratory studies showed that a recombinant *T. spiralis* galectin (rTsgal) has the function of agglomerating red blood cells from humans, rabbits, and mice, and this agglutination is inhibited by lactose. rTsgal specifically bound to the TLR-4 receptor on the gut epithelium, activated MAPK-NF-κB pathway, caused intestinal inflammation to worsen and pro-inflammatory cytokines expression to rise, and facilitated larval invasion [32,33]. Additionally, rTsgal also has the capacity to drive macrophage polarization towards M1 and to strengthen the macrophage’s ADCC killing activity on the NBLs [34]. The mixed immunization of mice with rTsgal and galactomannan induced a Th1/Th2-type immune response and local mucosal immune response, and produced an immune protective effect against *T. spiralis* infection. However, the infective larvae were not completely eliminated from the vaccinated animals after the *T. spiralis* challenge.

In this study, a novel galactoside-binding lectin family protein of *Trichinella spiralis* (TsGLFP; GenBank: XM_003380630.1) was retrieved from the *T. spiralis* draft genome [35]. TsGLFP belongs to the galectin family and has two carbohydrate recognition domains (CRDs). This study aimed to investigate TsGLFP’s basic biological features and role in macrophage M1 polarization and ADCC killing of larvae.

## 2. Results

### 2.1. Bioinformatics Analysis Results of TsGLFP

**Figure 1:**
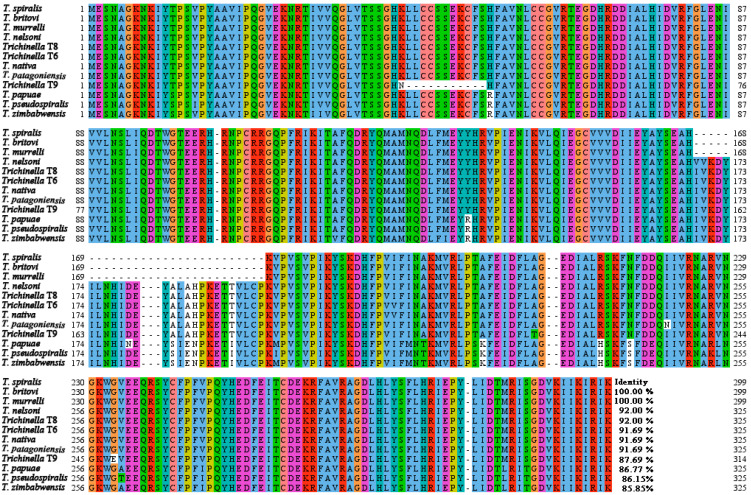
Multi sequence alignment of TsGLFP with galectin of different species/genotypes of the genus *Trichinella*. Amino acid sequence alignment of TsGLFP was performed using Jalview and MEGA 7.0. The same background color of amino acids at the same position among different *Trichinella* species/genotypes indicates that the amino acids are consistent, while a blank background color indicates that the amino acids are different. The figure shows that the amino acid sequence of TsGLFP is highly conserved among diverse species or genotypes within the genus *Trichinella*. The percentage of identity with TsGLFP is indicated by the number at the conclusion of each sequence.

**Figure 2:**
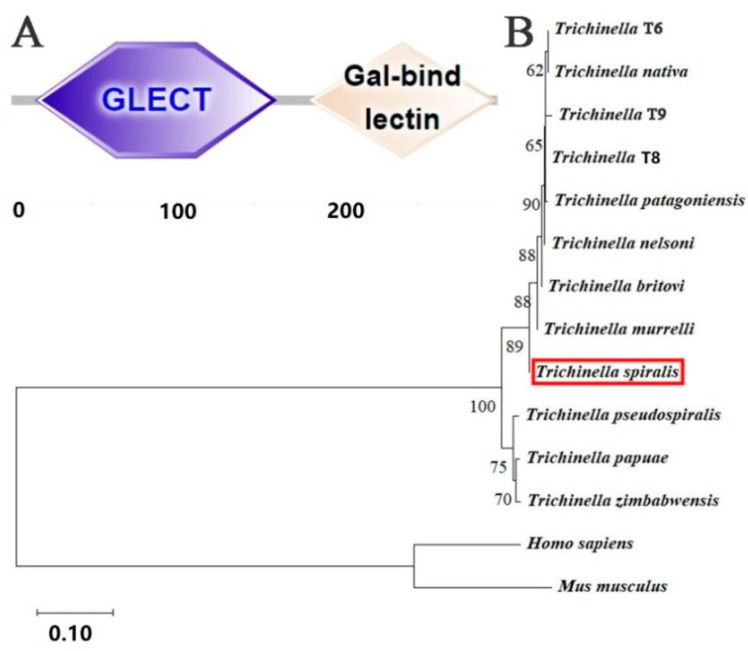
Structural domain and phylogenetic evolution tree of TsGLFP. (**A**): TsGLFP contains two sugar recognition domains localized at 16–161 aa and 185–296 aa, respectively. (**B**): A TsGLFP evolutionary tree among 12 diverse species or genotypes of the *Trichinella* genus was formed by using the neighbor-joining (NJ) method.

Bioinformatics analysis revealed that the complete cDNA sequence of TsGLFP was 900 bp, encoding 299 amino acids, and the molecular weight was 35 kDa with a pI of 7.15. The amino acid sequences of the TsGLFP had an identity of 100.00, 100.00, 92.00, 92.00, 91.69, 91.69, 91.69, and 87.69% with the galectin of the eight encapsulated *Trichinella* species/genotypes (*T. britovi*, *T. murrelli*, *T. nelsoni*, *Trichinella* T8, *Trichinella* T6, *T. nativa*, *T. patagoniensis*, and *Trichinella* T9). They also had an identity of 86.77, 86.15, and 85.85% with the galectin from three non-encapsulated *Trichinella* species (*T. papuae*, *T. pseudospiralis*, and *T. zimbabwensis*) (Figure 1). This TsGLFP protein is subcellularly localized within the cell. It has no signal peptides and no transmembrane regions. It also contains two structural domains at 16–161 aa and 185–296 aa that recognize carbohydrates: GLECT and Gal bind lectin domains (Figure 2A). The phylogenetic tree analysis revealed a monophyletic group of the genus *Trichinella*. Within the genus *Trichinella*, two distinct clades were exhibited: one was the clade of eight encapsulated species/genotypes (*T. nativa*, *Trichinella* T9, *Trichinella* T8, *T. murrelli*, *T. britovi*, *T. patagoniensis*, *Trichinella* T6, *T. nelsoni*), and the other was the clade of three non-encapsulated species (*T. papuae*, *T. pseudospiralis*, and *T. zimbabwensis*) (Figure 2B).

### 2.2. Expression, Purification, and Antigenicity Analysis of rTsGLFP

**Figure 3:**
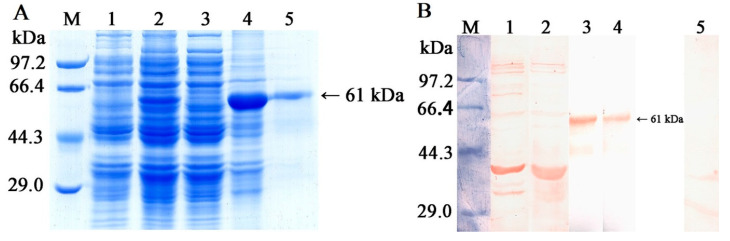
Identification of rTsGLFP. (**A**): SDS-PAGE analysis of rTsGLFP. Lane M: protein marker; Lane 1: lysate of bacteria carrying pGEX-4T-1/TsGLFP prior to induction; Lane 2: lysate of bacteria carrying pGEX-4T-1/TsGLFP following induction; Lane 3: supernatant of the recombinant bacterium lysate after induction; Lane 4: sediment of the recombinant bacterium lysate after induction; Lane 5: purified rTsGLFP (black arrow). (**B**): Western blotting analysis of rTsGLFP. The lysates of bacteria carrying pGEX-4T-1/TsGLFP prior to induction (Lane 1) and after induction (Lane 2) were not recognized by infection serum; Lane 3 and 4: purified rTsGLFP was recognized with anti-rTsGLFP serum (Lane 3) and anti-GST-tag serum (Lane 4; black arrow), but not by normal serum (Lane 5). Lane M: protein marker.

Following induction with IPTG, the fusion protein rTsGLFP tagged with the GST was produced in *E. coli* BL21 containing the recombinant plasmid pGEX-4T-1/TsGLFP. Once the rTsGLFP protein was successfully purified, SDS-PAGE analysis revealed a distinct single band, confirming a molecular weight of 61 kDa, aligning perfectly with its expected size (35 kDa for rTsGLFP plus 26 kDa for the GST tag) (Figure 3A). To evaluate the IgG antibody response triggered by rTsGLFP immunization, an ELISA was performed to measure the anti-rTsGFLP IgG titer, which registered at 1:10^5^ after the fourth round of immunizations. This finding indicates that rTsGLFP showcases strong antigenic properties. Additionally, a Western blot analysis confirmed the detection of rTsGLFP by anti-rTsGLFP serum, while it was not recognized by either the infection serum or normal murine serum (Figure 3B).

### 2.3. Transcription and Expression Levels of TsGLFP in Various Stages of Worms

**Figure 4:**
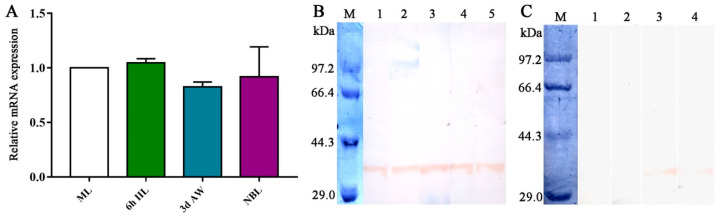
Transcription and expression levels of TsGLFP in various *T. spiralis* stages. (**A**): Transcriptional levels of TsGLFP in different *T. spiralis* phases. (**B**): Identification of native TsGLFP in crude antigens of *T. spiralis* MLs (Lane 1), IILs (Lane 2), 3 d AWs (Lane 3), 6 d AWs (Lane 4), and NBLs (Lane 5) by Western blotting with anti-rTsGLFP serum; Lane M: protein marker. (**C**): Western blotting identification of native TsGLFP in the ES antigens of various *T. spiralis* stages. The native TsGLFP protein in the ES antigens of MLs (Lane 1) and IILs (Lane 2) was not recognized by anti-rTsGLFP serum, but the native TsGLFP protein in the ES antigens of 3 d AWs (Lane 3) and 6 d AWs (Lane 4) was detected by anti-rTsGLFP serum; Lane M: protein marker.

The results of qPCR revealed that TsGLFP is transcribed in all worm stages of the nematode, and the transcription level showed no statistical difference at various stages (*H* = 9.971, *p* = 1) (Figure 4A). Western blot analysis showed that the natural TsGLFP of 35 kDa in the crude antigens of various worm stages was also identified by anti-rTsGLFP serum (Figure 4B). Moreover, Western blot analysis also showed that native TsGLFP was recognized in excretory–secretory (ES) antigens of 3 and 6 d AWs (Figure 4C).

### 2.4. Expression and Worm Tissue Localization of Natural TsGLFP by IIFT

**Figure 5:**
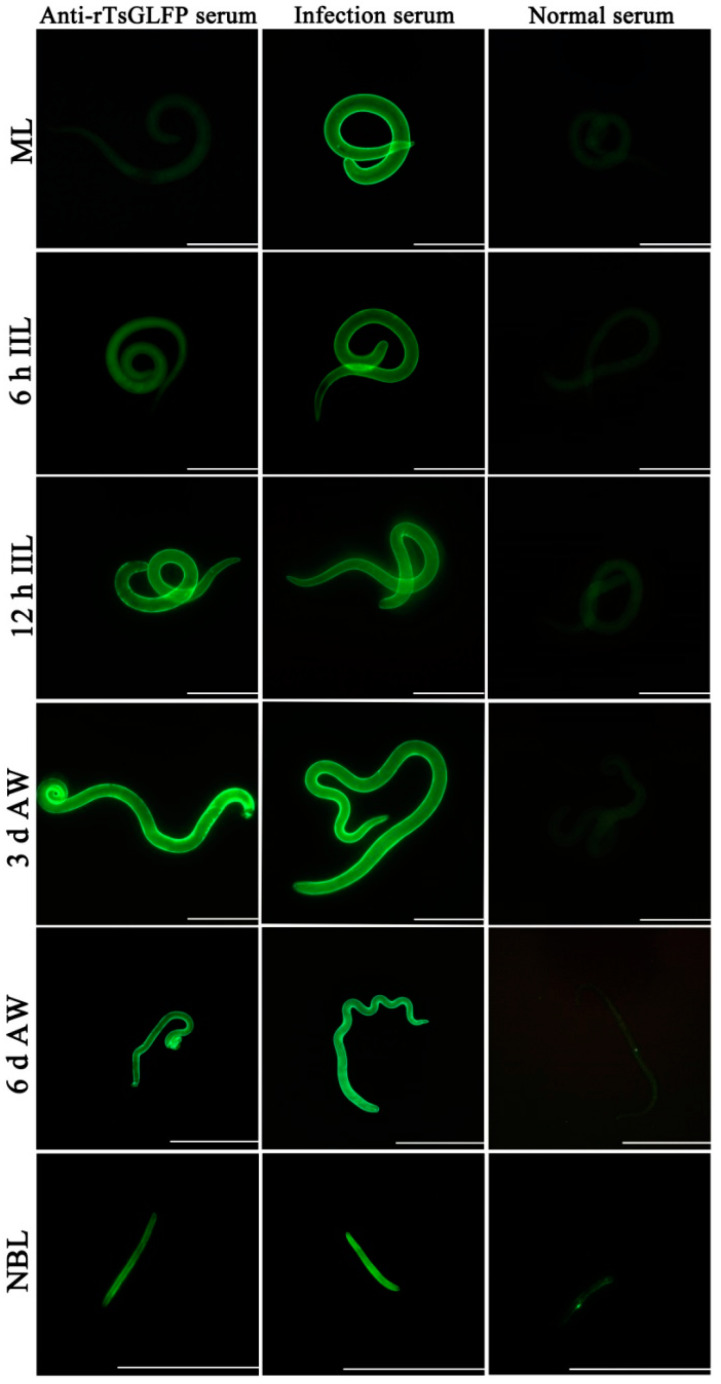
Expression of TsGLFP on the outer cuticle of various *T. spiralis* stages by IIFT. Intact whole worms were incubated with anti-rTsGLFP serum, and immunofluorescence was observed at the epicuticle of 12 h IILs, 3 and 6 d AWs, and NBLs, but not on the MLs and 6 h IILs. Furthermore, pre-immune normal mouse serum did not recognize the cuticle of the nematode. In the figure, the 6 d AWs scale bars are 500 μm, while the other stage worm scale bars are 200 μm.

**Figure 6:**
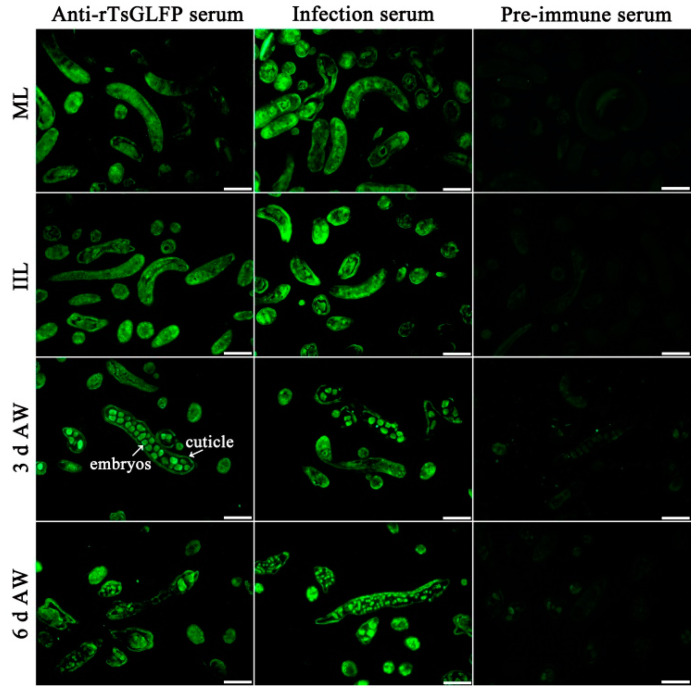
Distribution of TsGLFP within different stages of the worms. Immune fluorescence staining was observed in the cuticle and female intrauterine embryos. No immunostaining in worm cross-sections was detected by pre-immune normal serum as a negative control. Scale bars: 50 μm.

The results of IIFT with whole worms revealed that green immunofluorescence was observed on the outer cuticle of 12 h IILs, 3 and 6 d AWs, and NBLs by anti-rTsGLFP serum, but not on the surface of MLs and 6 h IILs (Figure 5). The IIFT with worm cross-sections showed that immunostaining is mainly localized in the worms’ cuticle, the whole section, and the embryos of the female adult worms (Figure 6).

### 2.5. rTsGLFP Hemagglutination Activity and Sugar Inhibition

**Figure 7:**
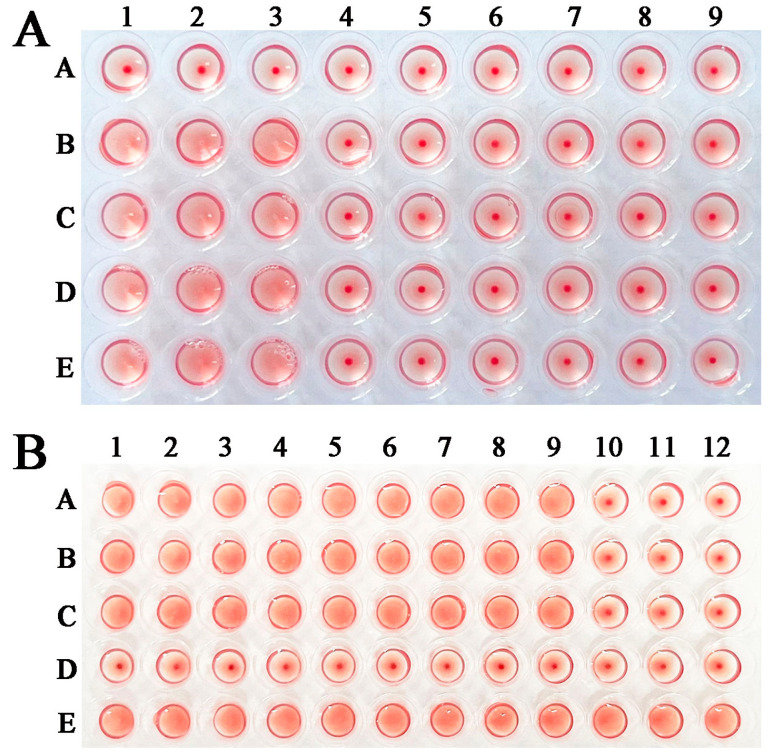
rTsGLFP hemagglutination activity and sugar inhibition. (**A**): Hemagglutination of murine erythrocytes with various concentrations of rTsGLFP. Lanes 1–3: rTsGLFP + erythrocytes; Lanes 4–6: GST + erythrocytes; Lanes 7–9: saline + erythrocytes; A–E: various concentrations of rTsGLFP (0, 50, 100, 200, and 400 μg/mL). (**B**): Inhibition of different carbohydrates on rTsGLFP hemagglutinating activity to murine erythrocytes. Lanes 1–3: glucose; Lanes 4–6: sucrose; Lanes 7–9: maltose; Lanes 10–12: lactose. A–C: different concentrations of carbohydrates (400, 200, and 100 mM). D: 100 mM various carbohydrates + erythrocytes (no rTsGLFP); E: 50 μg/mL rTsGLFP + erythrocytes (no carbohydrates).

rTsGLFP hemagglutination for mouse erythrocytes and sugar inhibition assays were performed. The results showed that rTsGLFP had the hemagglutinating erythrocyte role, and the minimal concentration of rTsGLFP agglutinating to erythrocytes was 50 μg/mL (Figure 7A). In the sugar inhibition test, lactose was the only carbohydrate that inhibited the agglutination of murine erythrocytes by rTsGLFP, and the minimal inhibitory dose of lactose was 100 mM (Figure 7B).

### 2.6. The Effect of rTsGLFP on the RAW264.7 Cellular Viability

**Figure 8:**
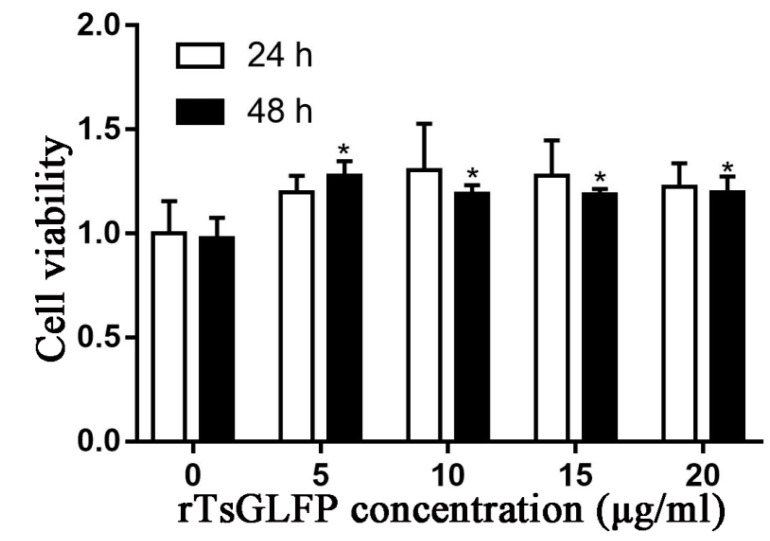
The effect of rTsGLFP on the viability of RAW264.7 macrophages. rTsGLFP (5, 10, 15, and 20 μg/mL) was co-incubated with RAW264.7 macrophages for 24 and 48 h, and the effect of rTsGLFP on the cell viability was assessed. The OD_450_ values were measured by the SpectraMax i3X (Molecular Devices, San Jose, CA, USA). The OD_450_ value served as the cell proliferation index. The data from three independent tests are exhibited as the mean ± SD. * *p* < 0.05 in comparison with the PBS group.

The cellular viability was assessed when the RAW264.7 cells were incubated with various doses of rTsGFLP (0–20 μg/mL) for 24 and 48 h. The results showed that 5–20 μg/mL rTsGFLP had no evident effects on cellular viability at 24 h (*F* = 1.787, *p* = 0.208), but the same concentration of rTsGLFP contrarily increased cell viability after incubation for 48 h (*F* = 8.522, *p* = 0.003). The results suggested that rTsGLFP (0–20 μg/mL) does not have any cytotoxicity to RAW264.7 cells; therefore, 15 μg/mL rTsGLFP and incubation for 24 h was used in the subsequent experiments (Figure 8).

### 2.7. rTsGLFP Drove the Macrophages’ M1 Polarization

**Figure 9:**
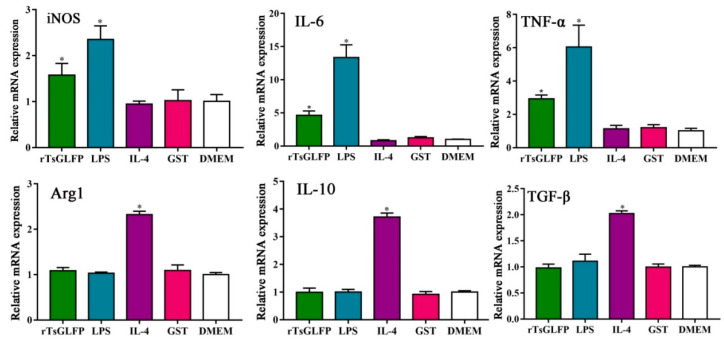
rTsGLFP drove the macrophages M1 polarization and increased M1 cytokine transcription levels. The mRNA expression of M1/M2 related genes in rTsGLFP-treated RAW264.7 macrophages was ascertained by qPCR. M1 macrophage-related genes are iNOS, IL-6, and TNF-α, M2 macrophage-related genes are Arg1, IL-10, and TGF-β. The mRNA expression levels of the genes were calculated with the Ct ^(2−ΔΔCt)^ method. GAPDH served as a housekeeping gene control. * *p* < 0.05 in contrast with the DMEM group.

To assess rTsGLFP’s role on macrophages’ polarization, RAW264.7 macrophages were simulated with rTsGLFP, and the subsequent mRNA levels of the M1 and M2 maker genes were ascertained using qPCR. The findings indicated a marked increase in the mRNA levels of M1 genes (inducible nitric-oxide synthase, iNOS; IL-6; and TNF-α) in RAW264.7 macrophages exposed to rTsGLFP, in contrast to the DMEM control (*F*_iNOS_ = 23.366, *p* = 0.009; *F*_IL-6_ = 39.228, *p* = 0.032; *F*_TNF-α_ = 33.982, *p* = 0.003) (Figure 9). However, there was no significant change in the mRNA levels of M2 genes (Arg1, IL-10, and TGF-β) when comparing to the DMEM group (*F*_Arg1_ = 165.029, *p* = 0.215; *F*_IL-10_ = 338.736, *p* = 0.934; *F*_TGF-β_ = 97.443, *p =* 0.747).

**Figure 10:**
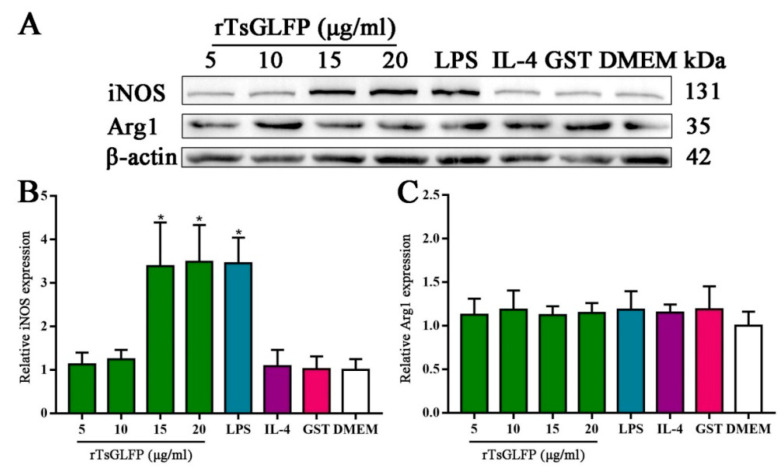
Expression level of M1/M2 markers in RAW264.7 macrophages stimulated by rTsGLFP. The expression of iNOS and Arg1 in RAW264.7 macrophages was ascertained by Western blotting after the macrophages were incubated with rTsGLFP (5–20 μg/mL) for 24 h. (**A**): Western blotting of iNOS and Arg1 expression in macrophages. β-actin was used as an internal control. (**B**): The intensity of the iNOS protein signal relative to the intensity of the β-actin control. (**C**): The intensity of the Arg1 protein signal relative to the intensity of the β-actin control. * *p* < 0.05 in contrast with the DMEM group.

Furthermore, Western blot analysis revealed that the protein expression of iNOS (an M1 marker) in RAW264.7 macrophages treated with 15 and 20 μg/mL of rTsGLFP was significantly elevated compared to the DMEM group (*F* = 14.303, *p* < 0.0001), whereas the Arg1 (M2) protein expression level was not noticeably changed in comparison to the DMEM group (*F* = 0.351, *p* = 0.917) (Figure 10). The results indicated that the rTsGLFP drove the macrophages’ M1 polarization and obviously induced the mRNA expression level of M1 cytokines (IL-6 and TNF-α).

### 2.8. rTsGLFP Activated the NF-κB Pathway

**Figure 11:**
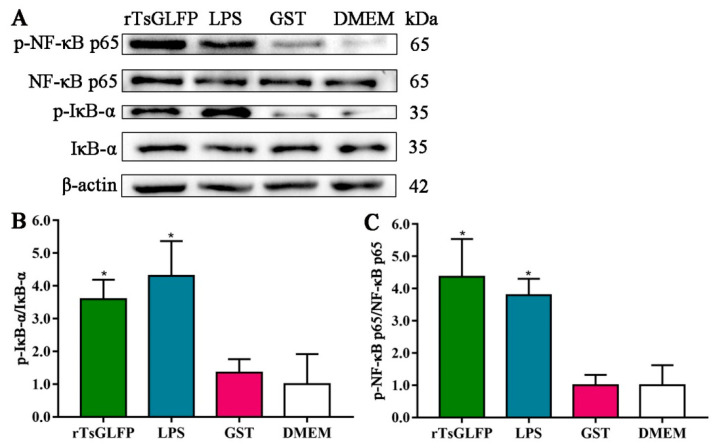
Expression level of p-IκB-α and p-NF-κB p65 in macrophages incubated with rTsGLFP. (**A**): The phosphorylation levels of NF-κB p65 and IκB-α in RAW264.7 macrophages stimulated by rTsGLFP. (**B**): The phosphorylation level of IκB-α. (**C**): The phosphorylation level of NF-κB p65. Each experiment had triplicate. * *p* < 0.01 in comparison with the DMEM group.

After the macrophages were stimulated with rTsGLFP, the expression levels of IκB-α and NF-κB p65 remained unchanged compared to the DMEM group (*p* > 0.05). In contrast, the expression levels of p-IκB-α and p-NF-κB p65 were clearly increased. The phosphorylation levels of IκB-α and NF-κB p65 were elevated by 2.59 and 3.36 times, respectively (*F*_p-IκB-α_ = 12.831, *p* = 0.004; *F*_p-NF-κB p65_ = 17.997, *p* < 0.0001), indicating that rTsGLFP induced RAW264.7 macrophages’ polarization towards M1 by activating the classical NF-κB pathway (Figure 11).

### 2.9. Inhibitor Suppressed the rTsGLFP-Driven Macrophages M1 Polarization and NF-κB Pathway Activation

**Figure 12:**
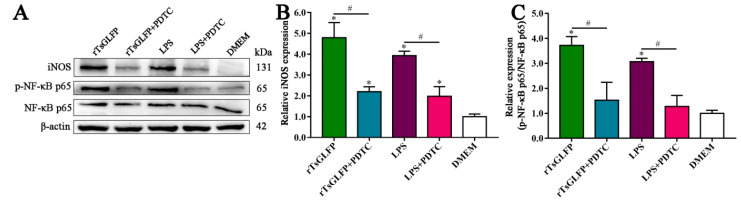
NF-κB pathway inhibitor suppressed rTsGLFP-driven M1 polarization and NF-κB pathway activation. (**A**): Western blot analysis of expression levels of iNOS, NF-κB p65, and p-NF-κB p65 in macrophages pretreated with PDTC. (**B**): iNOS expression level. (**C**): Phosphorylation level of NF-κB p65. * *p* < 0.05 relative to the DMEM group; ^#^ *p* < 0.01 compared between two groups.

When the macrophages were pretreated with the specific p-NF-κB p65 inhibitor PDTC, and then stimulated with rTsGLFP, compared with the rTsGLFP-only group, the iNOS expression level was decreased by 54.16% (*F* = 41.220, *p* < 0.0001). Similarly, the p-NF-κB p65 expression level was decreased by 59.14% (*F* = 24.868, *p* < 0.0001). These findings showed that the inhibitor PDTC definitely suppressed rTsGLFP-driven macrophages’ M1 polarization and NF-κB pathway activation, further confirming that the rTsGLFP-induced M1 polarization and expression level increase in iNOS was achieved by activating the NF-κB pathway (Figure 12).

### 2.10. Inhibitor Suppressed the Transcription Levels of iNOS and Pro-Inflammatory Cytokines in rTsGLFP-Treated Macrophages

**Figure 13:**
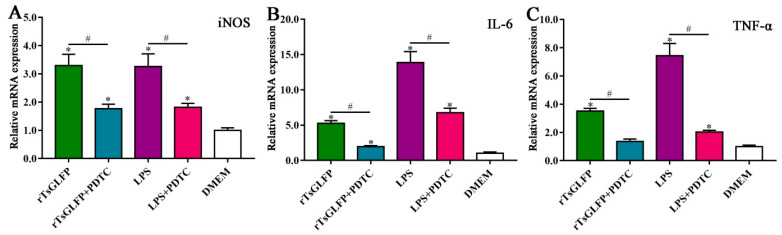
PDTC inhibited the transcription level of iNOS and pro-inflammatory cytokines in rTsGLFP-incubated macrophages. (**A**): Transcription level of iNOS. (**B**): Transcription level of IL-6. (**C**): Transcription level of TNF-α. * *p* < 0.05 contrast with the DMEM group; ^#^ *p* < 0.01 compared between two groups.

After the macrophages were pretreated with PDTC, the transcription level of iNOS and the pro-inflammatory cytokines IL-6 and TNF-α in rTsGLFP-treated macrophages was reduced by 46.24, 62.54, and 60.96%, respectively, compared to the rTsGLFP-only group (*F*_iNOS_ = 37.358, *p* < 0.0001; *F*_IL-6_ = 110.568, *p* = 0.006; *F*_TNF-α_ = 101.479, *p* = 0.001), further indicating that rTsGLFP-induced M1 polarization and pro-inflammatory cytokine responses are acquired via the NF-κB pathway (Figure 13).

### 2.11. rTsGLFP Increased the NO Production of Stimulated Macrophages

**Figure 14:**
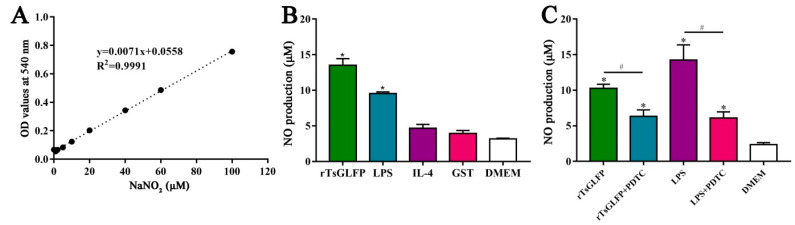
The NO production in supernatant of cultivated macrophages. (**A**): The standard curve of NO concentration. (**B**): NO production of macrophages cultivated with rTsGLFP, LPS, IL-4, or GST tag for 24 h. (**C**): NO production of PDTC-pretreated macrophages cultivated with rTsGLFP and LPS for 24 h. The macrophages were pre-incubated with PDTC for 24 h, then incubated with rTsGLFP for 24 h. LPS (200 ng/mL) was used as a positive control, while IL-4 (20 ng/mL) was used as a negative control. * *p* < 0.001 contrast with to the DMEM group; ^#^ *p* < 0.01 compared between two groups.

The Griess reaction was performed to assess the stimulating effect of rTsGLFP on the NO production of RAW264.7 macrophages. The standard curve of NO concentration was drawn based on the OD values at 540 nm of a serial concentration of NaNO_2_ (Figure 14A). The results revealed that the NO production in LPS-stimulated macrophages was evidently elevated compared to the DMEM group (*F* = 66.880, *p* < 0.0001). Still, the NO production in macrophages stimulated with IL-4 was not evidently changed relative to the DMEM group (*F* = 66.880, *p* = 0.082). However, after incubation with rTsGLFP, the NO production in rTsGLFP-stimulated macrophages was increased by 325.44% compared to the only-DMEM-medium group (*F* = 66.880, *p* < 0.0001) (Figure 14B). Additionally, after a specific NF-κB inhibitor (PDTC) was used, the NO production from the treated macrophages was also assayed, and the results showed that the NO production of PDTC-treated macrophages was decreased by 38.24% compared to the individual rTsGLFP group (*F* = 47.652, *p* = 0.002) (Figure 14C). The results further suggested that rTsGLFP drove the macrophages’ M1 polarization, and they indicated that rTsGLFP increased the NO production of M1 macrophages by activating the NF-κB pathway.

### 2.12. rTsGLFP Enhanced the Macrophages’ Cytotoxicity on NBL

**Figure 15:**
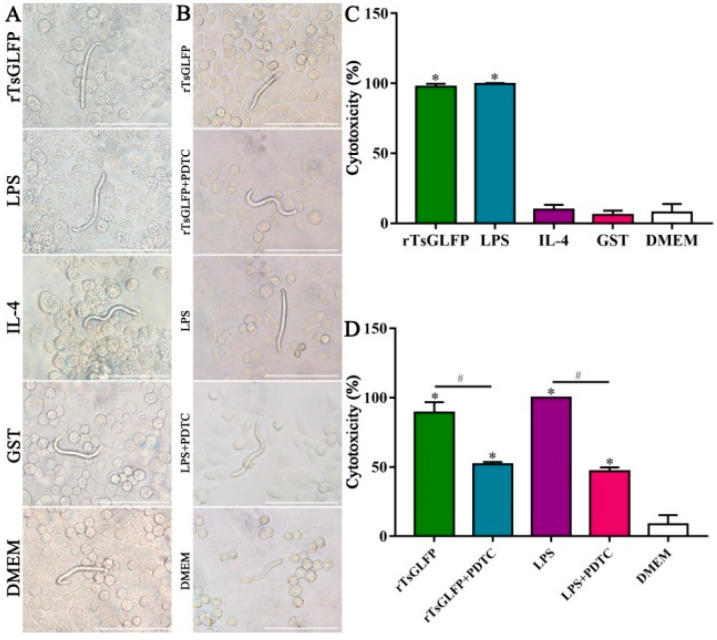
ADCC killing effects on the *T. spiralis* newborn larvae. (**A**): The cytotoxicity of macrophages stimulated with rTsGLFP for 36 h; (**B**): The cytotoxicity of macrophages pretreated with the specific NF-κB inhibitor PDTC for 2 h, and then stimulated with rTsGLFP for 24 h. (**C**,**D**): Cytotoxicity was assessed as the percent of dead larvae or NBLs with adherent macrophages to the number of total NBLs observed in each test. * *p* < 0.05 in comparison with the DMEM group; ^#^ *p* < 0.01 compared between two groups. Scale bars: 150 μm.

The results of the ADCC test revealed that after being cultured at 37 °C for 36 h, *T. spiralis*-infected murine sera mediated the macrophages’ adhesion to the NBLs and damage of the NBLs. In the group of rTsGLFP treated-macrophages, the cytotoxicity was increased by 11.31 times (*χ*^2^ = 823.724, *p* < 0.0001) and more macrophages were attached to the NBLs, compared to the DMEM group (Figure 15A,C). The cytotoxicity of LPS-treated macrophages was significantly elevated. In contrast, the NBL exhibited more activity with no apparent cytotoxic effects in the IL-4, GST, and DMEM groups. However, after the macrophages were pretreated with PDTC and then incubated with rTsGLFP for 36 h, the cytotoxicity was reduced by 41.62% compared to the rTsGLFP group alone (*χ*^2^ = 430.418, *p* < 0.0001) (Figure 15B,D). After the macrophages were pretreated with the p-NF-κB p65 inhibitor PDTC, and then used in the ADCC test, the NBL activity was significantly increased and fewer macrophages were attached to the NBLs, suggesting that the inhibitor PDTC suppressed the rTsGLFP-induced M1 polarization and NF-κB pathway activation. These findings demonstrated that the rTsGLFP enhanced the macrophages’ cytotoxicity which was dependent on the macrophage M1 polarization and NF-κB pathway activation.

## 3. Discussion

Galectin typically contains one to two carbohydrate recognition domains (CRDs) and has a high affinity for β-galactoside [36]; it plays an essential role in cell differentiation, proliferation, apoptosis, adhesion, and migration [37]. Galectin secreted by *Brugia malayi* activated macrophages, induced Th1 cell apoptosis, and triggered a type 2 immune response in chronic lymphatic filariasis [29]. In the process of *Schistosoma mansoni* infection, galectin-3 plays a role in maintaining splenic construction, controlling cell apoptosis, macrophage activity, B-cell differentiation, and plasmacytogenesis [38].

In this study, we constructed the pGEX-4T-1/TsGLFP expression plasmid, which was transformed into the *Escherichia coli* BL21 strain. rTsGLFP protein was induced, expressed and purified. The TsGLFP is identified as galectin because of its two CRD structure domains. The agglutination activity of rTsGLFP was also ascertained in the present study. The results showed that rTsGLFP was capable of agglutinating mouse red blood cells and this agglutination activity was inhibited by lactose. The rTsGLFP specially binding with lactose might be involved with its two CRD structure domains [39]. The sequence alignment showed that TsGLFP had a high amino acid sequence identity of 85.85–100% with the galectin of the 11 species/genotypes of the genus *Trichinella*, suggesting that GLFP might have a similar function in various *Trichinella* species/genotypes [40]. Immunization of mice with rTsGLFP triggered a specific humoral anti-rTsCTL IgG antibody response, and the serum anti-rTsCTL IgG antibody titer was up to 1:10^5^, indicating that rTsGLFP had a good immunogenicity. Western blotting revealed that rTsGLFP could be recognized by anti-rTsGLFP immune serum. The transcription and expression levels of TsGLFP were assessed by qPCR and Western blot. The results revealed that TsGLFP was transcribed and expressed in various *T. spiralis* worm phases (MLs, IILs, AWs, and NBLs). Natural TsGLFP was also identified in the ES antigens of 3 d and 6 d AWs. The IIFT results showed that TsGLFP is mainly located in the cuticle of 12 h IILs, 3 and 6 d AWs, NBLs, and in the female embryos. The results suggested that TsGLFP is a necessary protein for development, reproduction, and survival in the lifecycle of this nematode. *T. spiralis* surface antigens, as the principal target antigens, are directly contacted by the host immune system, and may play a main role in worm immune evasion, development, and survival in the host [41,42,43].

When *T. spiralis* larvae penetrate into the enteral epithelium, intestinal larva invasion leads to an evident enteral mucosal inflammation during the enteral stage of *T. spiralis* infection [44]. Macrophages exist in many tissues; those from intestinal mucosa have different phenotypes, which together maintain tolerance to gut microbiota and oral antigens. According to functional differences, macrophages are divided into M1 and M2 types [45,46]. M1 macrophages play a pro-inflammatory role, while M2 macrophages predominantly exert anti-inflammatory effects [47,48]. M1 macrophages respond to Toll-like receptor stimulation or pro-inflammatory cytokine induction and are considered significantly pro-inflammatory and parasite-killers. M2 macrophages respond to type 2 inflammatory factors such as IL-4, which is believed to be associated with tissue repair and parasite infection [49,50]. In the process of parasitic infection, macrophages undergo dynamic changes from the M1 type to the M2 type depending on the duration of infection [51]. Polysaccharides on the worm surface cause CD4^+^ cells to polarize towards M2 by binding to host macrophage C-type lectins or Toll-like receptors [52]. We speculate that rTsGLFP might activate macrophages’ polarization towards M1, cause intestinal inflammation, and promote parasite expulsion from the gut.

Our results revealed that after the mouse macrophages RAW264.7 were incubated with rTsGLFP, the mRNA level of the M1 genes (iNOS, IL-6, and TNF-α) was significantly increased; whereas the mRNA level of M2 genes (Arg1, IL-10, and TGF-β) was not evidently changed. Furthermore, Western blotting results exhibited that the iNOS (M1) expression level in rTsGLFP-treated cells was significantly increased, while the Arg1 (M2) expression level was not definitively changed. The results suggested that TsGLFP drove the macrophages’ M1 polarization and clearly raised the expression level of M1 pro-inflammatory cytokines (IL-6 and TNF-α), which enhanced the intestinal inflammatory reaction [33,53].

The inflammatory signaling pathway involves activating the nuclear transcription factor NF-κB in the cell nucleus, which results in the production of downstream inflammatory factors, cellular autophagy, and apoptosis [54]. NF-κB is a trimer consisting of IκB, p50, and p65. p65 is activated into phosphorylated p65 and then enters the nucleus, leading to the expression of diverse cytokines (inflammatory factors and adhesion molecules), thereby taking part in several inflammatory diseases [55]. Previous studies indicated that the NF-κB pathway was activated, and TLR-4 and TLR-9 expression was increased in the intestinal tissues of *T. spiralis*-infected mice [56]. To investigate the mechanism of macrophages’ M1 polarization and to assess whether rTsGLFP activates the NF-κB signaling pathway, the NF-κB pathway protein expression level in rTsGLFP-treated macrophages was assessed by Western blot. The results revealed that the expression levels of p-IκB-α and p-NF-κB p65 were undoubtedly increased after macrophages being incubated with rTsGLFP for 24 h. The phosphorylation levels of IκB-α and NF-κB p65 were elevated by 2.59 and 3.36 times, respectively, compared to the DMEM control group. When the macrophages were pretreated with the specific p-NF-κB p65 inhibitor PDTC, and then stimulated with rTsGLFP, compared with the only rTsGLFP group, the expression level of iNOS and p-NF-κB p65 was decreased by 54.16 and 59.14%, respectively, suggesting that the inhibitor PDTC noticeably suppressed rTsGLFP-driven macrophages’ M1 polarization and NF-κB pathway activation. These findings confirmed that rTsGLFP drove macrophages’ M1 polarization by activating the NF-κB pathway. The results are similar to previous studies on another *T. spiralis* galectin (Tsgal). rTsgal binding with TLR-4 on the gut epithelium activated the MAPK-NF-κB pathway, increased the expression levels of TLR-4 and pro-inflammatory cytokines, deteriorated enteral inflammation, and mediated larva invasion [33]. rTsgal also alleviated DSS-induced murine colitis by inhibiting the TLR-4 (TLR-2)/MyD88/NF-κB signaling pathway, reducing excessive inflammatory responses, improving inflammatory cell infiltration and tissue damage [57].

NO is produced by inducible nitric-oxide synthase (iNOS) via converting L-arginine into citrulline by using NADPH and oxygen [58]. The polysaccharide has been used as a macrophage activator and is regarded as the most powerful NO pathway stimulator [59]. Polysaccharides may stimulate the TLR2- and TLR4-mediated MAPKs and NF-κB pathways, which in turn may enhance macrophage production of NO, reactive oxygen species (ROS), TNF-α, and IL-6 [60]. In the current investigation, NO generation in rTsGLFP-treated macrophages was also assessed. The finding showed that rTsGLFP clearly increased the NO synthesis and secretion because of its activating macrophage M1 polarization. Our results also showed that the larval killing ability of rTsGLFP-treated macrophages through ADCC was also distinctly strengthened. The p-NF-κB p65 specific inhibitor PDTC suppressed the polarization of macrophages towards M1 and reduced the expression of iNOS as well as NO, and the pro-inflammatory cytokines IL-6 and TNF-α, and decreased and abrogated the rTsGLFP-enhanced macrophages’ cytotoxicity killing NBLs. The immune response elicited at the intestinal phase of *T. spiralis* infection not only was directed toward the adult worms but also towards the NBLs, as the cytotoxic activity against NBLs was significantly enhanced [61]. Our results further verified that rTsGLFP induced macrophage M1 polarization and strengthened its cytotoxicity on NBLs via the NF-κB signaling pathway [34]. Previous studies revealed that macrophages are able to directly damage *T. spiralis* NBLs by mediating ADCC and producing NO [62,63]. The results indicated that by inducing macrophage M1 polarization, rTsGLFP enhanced the killing ability of macrophage-mediated ADCC on NBLs, and the M1-type macrophages released pro-inflammatory cytokines (TNF-α and IL-6) as well as natural killing molecules (NO). Nevertheless, the detailed mechanism of rTsGLFP driving macrophage M1 polarization, such as the kind and features of the galectin receptor or ligand on macrophages, needs to be characterized in future experiments.

## 4. Materials and Methods

### 4.1. Ethics Statement

This research was carried out according to the National Guidelines for Experimental Animal Welfare (Minister of Science and Technology, People’s Republic of China, 2006). All animal experiment protocols in the present research were authorized by the Zhengzhou University Life Science Ethics Committee (No. ZZUIRB GZR 2022-1317).

### 4.2. Parasites, Experimental Animals and Cells

The present investigation employed the *Trichinella spiralis* isolate (ISS534), which was obtained from a naturally infected pig in the Henan Province of China. Prior to preservation, the isolate was passaged in BALB/c mice [8]. BALB/c mice were purchased from Henan Provincial Experimental Animal Center (Zhengzhou, China). Mouse leukemia cells of the monocyte/macrophage line RAW264.7 were gained from the Cell Bank of Chinese Academy of Sciences (Shanghai, China) [34].

### 4.3. Collection of Various T. spiralis Stages and Preparation of Crude and ES Antigens

*T. spiralis*-infected mouse skeletal muscles at 42 days post-infection (dpi) were digested by artificial digestion to collect the MLs [40,64]. The IILs were recovered from infected mouse gut at 6 h post-infection (hpi). AWs were extracted at 3 and 6 dpi from the mouse gut. Female AWs were cultivated in RPMI-1640 supplemented 10% fetal bovine serum (FBS; Gibco, Waltham, MA, USA) at 37 °C in 5% CO_2_ for 24 h, and the newborn larvae (NBLs) were recovered [65]. As previously reported, excretory–secretory (ES) proteins from AWs and the somatic crude proteins of MLs, IILs, AWs, and NBLs in worms were produced [66].

### 4.4. Bioinformatics Analysis

The complete-length cDNA sequence of the TsGLFP gene was acquired from GenBank (XM_003380630.1). The TsGLFP physical and chemical features (signal peptide, transmembrane regions, subcellular localization, tertiary structure, and functional domain) were predicted using the ProtParam tool (https://web.expasy.org/protparam/, accessed on 3 October 2024) [67,68]. The protein molecular weight and isoelectric points of TsGLFP were estimated by using ExPASy online software (https://web.expasy.org/protparam/, accessed on 3 October 2024). The amino acid sequence of TsGLFP was compared with galectin from other organisms by using BioEdit software, version 7.7. The GenBank accession numbers of galectin from other organism were as follows: *T. britovi* (KRY47073.1), *T. murrelli* (KRX44687.1), *Trichinella* T6 (KRX71482.1), *T. nelsoni* (KRX16983.1), *Trichinella* T8 (KRZ96027.1), *T. nativa* (KRZ50480.1), *T. patagoniensis* (KRY20697.1), *T. papuae* (KRZ70401.1), *T. pseudospiralis* (KRX96821.1), *T. zimbabwensis* (KRZ11582.1), *Trichinella* T9 (KRX61370.1), *Mus musculus* (NP 001399591.1), and *Homo sapiens* (4HAN_A). The multi-sequence alignment and phylogenetic tree construction of TsGLFP were carried out by using Jalview and MAGA 7.0 with the neighbor-joining (NJ) method.

### 4.5. Cloning, Expression, and Identification of rTsGLFP

The total RNAs of *T. spiralis* MLs were isolated using TRIzol reagent (Invitrogen, Waltham, MA, USA) and reversely transcribed into the cDNA. The overall length of the cDNA sequence of the TsGLFP gene (GenBank: XM_003380630.1) was amplified by PCR using specific primers with BamH I and Xho I restriction sites (in bold and shadowed) as follows: 5′-CG**GGATCC**ATGGAATCGAATGCTGGAAA-3′ and 5′-CGC**CTCGAG**TCACTTGATACGTATTTTG-3′. After being amplified, the PCR products were cloned into pGEX-4T-1 to construct pGEX-4T-1/TsGLFP, which was transformed into the *E. coli* BL21 strain (DE3) (Novagen, Madison, WI, USA). Then, 0.1 mM isopropyl β-D-1-thiogalactopyranoside (ITPG) was used to induce the expression of rTsGLFP at 16 °C for 24 h [32], and the GST-Sefinose (TM) Resin 4FF (Settled Resin) (Sangon Biotech., Shanghai, China) was applied for the purification of rTsGLFP. The expression and antigenicity of the rTsGLFP were analyzed by SDS-PAGE and Western blot.

### 4.6. Preparation of Anti-rTsGLFP Serum

Twenty BALB/c mice were given a subcutaneous immunization with 20 µg rTsGLFP emulsified with complete Freund’s adjuvant for each mouse. After two weeks, the mice were given three booster injections of 20 μg of rTsGLFP emulsified with incomplete Freund’s adjuvant [69]. Two weeks after the fourth immunization, the vaccinated mice’s tail blood was obtained to isolate anti-rTsGLFP serum. The IgG level of the anti-rTsSPc antibody was measured by ELISA with rTsGLFP as a coating antigen.

### 4.7. SDS-PAGE and Western Blotting

The worm proteins consisted of crude and ES antigens from various stages of *T. spiralis* worms (MLs, IILs, 3 and 6 d AWs, or NBLs) and rTsGLFP. The proteins were separated using a 10% separation gel on SDS-PAGE and then transferred onto polyvinylidene fluoride (PVDF) membrane (Millipore, Burlington, MA, USA) in the wet transfer cell (Bio-Rad, Hercules, CA, USA) at 0.4 A for 30 min [70]. After blocking the membrane for an hour at 37 °C with 5% skim milk in Tris-buffered saline containing 0.05% Tween-20 (TBST), the membrane was sliced into strips. The strips were incubated at 4 °C overnight with 1:100 dilutions of different sera, including anti-rTsGLFP serum, *T. spiralis*-infected murine serum collected at 35 dpi, and normal mouse serum; anti-GST tag antibody served as negative control (servicebio, Wuhan, China). After washing, the strips were incubated with 1:10,000 dilutions of HRP-goat anti-mouse IgG conjugate (SouthernBiotech, Birmingham, AL, USA) at 37 °C for 1 h. The color was developed by using 3-amino-9-ethylcarbazole (AEC; Solarbio, Beijing, China) and stopped by washing the membrane with deionized water [71].

### 4.8. Real-Time Quantitative PCR (qPCR) Test

Total RNAs from various stages of *T. spiralis* worms (MLs, IILs, 3 d AWs, and NBLs) were extracted using TRIzol reagent (Invitrogen). The TsGLFP mRNA expression level at various stages was assessed by qPCR as reported previously [72,73]. The TsGLFP-specific primers for qPCR were 5′-CGGGATCCATGGAATCGAATGCTGGAAA-3′ and 5′-CGCCTCGAGTCACTTGATACGTATTTTG-3′. The relative TsGLFP mRNA expression level was calculated by subtracting the mRNA expression level of the *T. spiralis* housekeeping gene GAPDH (GenBank: AF452239) [74] and then calculated on the basis of the comparative Ct (2^−ΔΔCt^) method [75]. Each test had three replicates.

In addition, RAW264.7 macrophages were treated using 15 μg/mL rTsGLFP at 37 °C for 24 h, 200 ng/mL lipopolysaccharide (LPS) was used as the M1 positive control, 20 ng/mL IL-4 was used as the M2 positive control, and 15 μg/mL GST as the tag control. The mRNA expression of the M1 marker (inducible nitric-oxide synthase, iNOS) and cytokines (IL-6 and TNF-α) and the M2 marker (Arg1) and cytokines (IL-10 and TGF-β) was also determined by qPCR. The primer sequences used for qPCR amplification are displayed in Table 1 [76]. Moreover, after RAW264.7 macrophages were pretreated by Pyrrolidinecarbodithioic acid (PDTC), a specific NF-κB inhibitor which can permeate the cell membranes and inhibits the activation of NF-κB [77,78,79], the mRNA expression of the M1 marker (iNOS) and cytokines (IL-6 and TNF-α) was assessed by qPCR [33]. Briefly, the PDTC storage solution was initially prepared in accordance with the manuals: one milliliter of PBS is used to directly dissolve 8.2145 mg of PDTC powder to create a 50 mM concentration storage solution. The macrophages were treated with PDTC (150 μM) for 2 h, then incubated with rTsGLFP, LPS, or GST tag for 24 h, and the mRNA level of iNOS, IL-6, and TNF-α was detected by qPCR.

### 4.9. Indirect Immunofluorescence Test (IIFT)

The expression and tissue localization of natural TsGLFP at different *T. spiralis* worm stages (MLs, 6 and 12 h IILs, 3 and 6 d AWs, and NBLs) was investigated by IIFT with intact worms and their cross-sections as previously reported [80]. Following their recovery and fixation in 4% paraformaldehyde, the fresh entire *T. spiralis* worms were embedded in paraffin and microtomed to create 2 µm-thick cross-sections [81]. Using 5% goat serum, the cross-sections and intact worms were blocked for 2 h at 37 °C. Following three washes in PBST, the samples were treated with various sera (1:10 dilutions of anti-rTsGLFP serum, *T. spiralis*-infected mouse serum, and pre-immune normal mouse serum) at 37 °C for 2 h. They were incubated with Alexa Fluor 488 conjugated anti-mouse IgG (1:100; Sangon Biotech., Shanghai, China) after additional washes. Finally, the entire worms and their cross-sections were examined under a fluorescence microscope (Olympus, Tokyo, Japan). When the bright green fluorescence on the surface of entire worms and their cross sections was seen, the result was judged as positive.

### 4.10. Erythrocyte Agglutination Test and Sugar Inhibition Test

Mouse blood samples were used in this investigation. After centrifuging the erythrocytes for 5 min at 350× *g*, they were washed three times with 0.9% sterile saline solution (pH 7.0). The erythrocytes were then resuspended in 2% saline solution. A hemagglutination activity assay of rTsGLFP was performed in accordance with earlier reports [70,82,83]. Briefly, 25 μL of varying rTsGLFP dilutions (0–400 μg/mL) was introduced onto the microtitration plate. Subsequently, 25 μL of 2% erythrocyte suspensions was added to every well. After 30 min of room temperature incubation, the plate was examined using a microscope to detect the whole erythrocyte agglutination. The lowest dose of rTsGLFP required to cause agglutination was noted. There were three copies of each experiment.

The sugar inhibition test was carried out as described before [40,84]. In this test, four different carbohydrates (lactose, sucrose, glucose, and maltose) were employed. In total, 25 μL of rTsGLFP (50 μg/mL) was added to the V-shape plate, and 25 μL different dilutions of carbohydrates (100–400 mM) were subsequently added into the well. To evaluate the inhibitory effects of the four carbohydrates on hemagglutination, 2% suspensions of murine erythrocytes were added and incubated for an additional hour at room temperature.

### 4.11. CCK8 Assay of RAW264.7 Cell Viability

The Cell Counting Kit-8 (CCK-8; Epizyme Biotech., Shanghai, China) was used to measure the effect of rTsGFLP on RAW264.7 macrophage viability [33,85,86]. In short, 1 × 10^5^ RAW264.7 cells were seeded in a 96-well plate and cultured in high glucose Dulbecco’s modified Eagle medium (DMEM; Servicebio, Wuhan, China) with 100 U/mL penicillin, 100 μg/mL streptomycin, and 10% fetal bovine serum (FBS; HyClone, Logan, UT, USA). The macrophages and different dosages of rTsGFLP were co-cultured at 37 °C for 24 and 48 h. After that, each well of the plate received 10 μL of CCK-8 reagent solutions, and the plates were then incubated for 2 h. The absorbance was measured at 450 nm by a multi-mode reader [87]. Cell viability was shown as the cellular survival rate based on the following formula: cellular survival rate = (OD values of test group − OD values of blank control)/(OD values of DMEM control group − OD values of blank control) × 100%.

### 4.12. Western Blotting of Expression of M1/M2 Markers, Cytokines, and the NF-κB Pathway in rTsGLFP-Stimulated Macrophages

Cellular soluble crude proteins from rTsGFLP-treated RAW264.7 cells were prepared in ice-cold cell lysis buffer (Beyotime, Shanghai, China) containing 1 mM phenylmethylsulfonyl fluoride (PMSF) [88]. Cellular protein concentration was assayed with a BCA assay kit (Solarbio, Beijing, China). Cellular proteins were separated by 10% SDS–PAGE, transferred onto PVDF membranes, and blocked with 5% skimmed milk TBST for 1 h at 37 °C. The membrane was cut into strips, and each strip was incubated with primary antibodies in TBST overnight at 4 °C as follows: anti-iNOS antibody (1:10,000; Abcam, London, UK), anti-Arg1 antibody (1:1000; Proteintech, Tokyo, Japan), anti-β-actin (1:1000; servicebio), anti-IκB-α antibody (1:1000; Abcam), anti-p-IκB-α antibody (1:1000; Abcam), anti-NF-κB p65 antibody (1:1000; Abcam), or anti-p-NF-κB p65(1:1000; Abcam). After washes with TBST, the strips were incubated at 37 °C for 1 h with HRP-anti-mouse IgG or HRP-anti-rabbit IgG conjugate (1:10,000; Southern Biotech). The Omni-ECLTm reagents (Epizyme, Shanghai, China) were used to visualize the reactive bands, and the relative intensity of each band was evaluated by using ImageJ software, version 1.8 (National Institutes of Health, Bethesda, MD, USA) [70,89].

### 4.13. Assay of Nitric Oxide (NO)

NO functions as a pleiotropic regulator in various biological pathways to defend the causative agents or foreign substances, making it a significant inflammatory mediator. NO secretion from macrophages was ascertained by testing the accumulated nitrite released into culture medium, relying on the Griess reaction [90]. In short, 6-well plates were seeded with RAW264.7 macrophages (1 × 10^5^ cells/well), then cultivated for 24 h. The cells were cultured in culture medium supplemented with rTsGLFP after the media was exchanged. The LPS (200 ng/mL)/IL-4 (20 ng/mL) was applied as an M1/M2 positive control, while the DMEM medium acted as a negative control, respectively. After being stimulated for 24 h, the NO generation in the culture medium was assayed with a NO assay kit (Beyotime) based on the Griess reaction. Following incubation at room temperature for 5 min, the solution’s absorbance at 540 nm was assayed by a multi-mode reader. The standard curve was drawn according to various concentrations of NaNO_2_. Each assay was performed in triplicate [91].

### 4.14. Antibody-Dependent Cell-Mediated Cytotoxicity (ADCC) Test

The macrophage killing of NBLs via ADCC was conducted as previously mentioned [63,92]. The RAW264.7 macrophages (9.6 × 10^5^ cells/well) were cultured in a 96-well plate and pre-incubated with various proteins (rTsGLFP, LPS, IL-4, GST tag, or only DMEM medium) for 36 h. Following medium renewal, 200 NBLs were added into the medium containing 1:50 dilutions of serum from mice infected with *T. spiralis*, and the mixture was incubated at 37 °C for 36 h. Since the NBLs were cultivated with various groups of macrophages and infection serum, larval viability was ascertained according to larval morphology and activity. The percentage of dead NBLs or NBLs with adhering macrophages to the total NBLs seen in each test was referred to as the cytotoxicity [8,80]. Furthermore, to investigate the effect of the NF-κB-specific inhibitor PDTC on the macrophages’ cytotoxicity, RAW264.7 macrophages were pretreated with PDTC, and then incubated with rTsGLFP and used for the ADCC test [33,93].

### 4.15. Statistical Analysis

All the data were analyzed by SPSS 21.0 software and the results are shown as the mean ± standard deviation (SD). The Kruskal–Wallis test was employed to analyze the variations in relative TsGLFP mRNA expression levels at different stages of the worm’s life. The Chi-square test was used to compare the variations of cytotoxicity in various groups. One-way ANOVA was employed to analyze the transcription and expression levels of iNOS, Arg1, and cytokines, NO production, and the phosphorylation level of the NF-κB signaling pathway. *p* < 0.05 was considered statistically significant.

## 5. Conclusions

TsGLFP was transcribed and expressed at diverse *T. spiralis* lifecycle stages, and it was principally localized on the cuticle and around the female embryos of the nematode. rTsGLFP had a good antigenicity, it also had hemagglutination activities on murine erythrocytes, and lactose suppressed rTsGLFP’s hemagglutinating function. rTsGLFP drove macrophage M1 polarization, evidently increased the expression level of NO and pro-inflammatory cytokines (IL-6 and TNF-α), and strengthened the macrophage cytotoxicity on NBL via the NF-κB signaling pathway. These findings indicated that rTsGLFP plays an important function in *T. spiralis* infection.

## Figures and Tables

**Table 1:** Specific primer sequences of murine macrophage markers and cytokines for qPCR.

Genes	Forward (5′-3′)	Reverse (5′-3′)
iNOS	GAGACAGGGAAGTCTGAAGCAC	CCAGCAGTAGTTGCTCCTCTTC
Arg1	CATTGGCTTGCGAGACGTAGAC	GCTGAAGGTCTCTTCCATCACC
IL-6	TACCACTTCACAAGTCGGAGGC	CTGCAAGTGCATCATCGTTGTTC
TNF-α	TCTTCTCATTCCTGCTTGTGG	CACTTGGTGGTTTGCTACGA
IL-10	CGGGAAGACAATAACTGCACCC	CGGTTAGCAGTATGTTGTCCAGC
TGF-β	TGATACGCCTGAGTGGCTGTCT	CACAAGAGCAGTGAGCGCTGAA
GAPDH	GGTTGTCTCCTGCGACTTCA	TGGTCCAGGGTTTCTTACTCC

## Data Availability

The original contributions presented in the study are included in the article, further inquiries can be directed to the corresponding authors.
